# A Wavelet-Based Learning Model Enhances Molecular Prognosis in Pancreatic Adenocarcinoma

**DOI:** 10.1155/2021/7865856

**Published:** 2021-10-16

**Authors:** Binhua Tang, Yu Chen, Yuqi Wang, Jiafei Nie

**Affiliations:** Epigenetics & Function Group, Hohai University, Jiangsu 213022, China

## Abstract

Genome-wide omics technology boosts deep interrogation into the clinical prognosis and inherent mechanism of pancreatic oncology. Classic LASSO methods coequally treat all candidates, ignoring individual characteristics, thus frequently deteriorating performance with comparatively more predictors. Here, we propose a wavelet-based deep learning method in variable selection and prognosis formulation for PAAD with small samples and multisource information. With the genomic, epigenomic, and clinical cohort information from The Cancer Genome Atlas, the constructed five-molecule model is validated via Kaplan-Meier survival estimate, rendering significant prognosis capability on high- and low-risk subcohorts (*p* value < 0.0001), together with three predictors manifesting the individual prognosis significance (*p* value: 0.0012~0.024). Moreover, the performance of the prognosis model has been benchmarked against the traditional LASSO and wavelet-based methods in the 3- and 5-year prediction AUC items, respectively. Specifically, the proposed model with discrete stationary wavelet base (bior1.5) overwhelmingly outperformed traditional LASSO and wavelet-based methods (AUC: 0.787 vs. 0.782 and 0.721 for the 3-year case; AUC: 0.937 vs. 0.802 and 0.859 for the 5-year case). Thus, the proposed model provides a more accurate perspective, but with less predictor burden for clinical prognosis in the pancreatic carcinoma study.

## 1. Introduction

Pancreatic adenocarcinoma (PAAD) cancer is one of the leading causes of global cancer-related mortality, but until now, the diagnosis and prognosis are still hysteretic with its death toll approaching the diagnosed cases [1]. Pancreatic ductal adenocarcinoma (PDAC) is the most common subtype of pancreatic cancer, named for its histological similarity to ductal cells [2, 3]. Quite a few developed countries have 3 to 4 times higher pancreatic cancer incidence than developing nations, with the highest rates in Europe, North America, Australia, and New Zealand. Notably, in many EU countries, pancreatic cancer is overtaking breast cancer as the third leading cause of cancer death [4].

In previous diagnosis and prognosis studies, Park et al. inferred prognostic markers from patient-specific gene relevance networks and identified diverse prognostic gene pairs [5]. Wang and Wei proposed a multivariate mixed model (IMIX) framework to screen genes and CNV related to the prognosis of pancreatic cancer, and the key genes identified were positively correlated with CNV status [6]. Nikolova et al. proposed a Bayesian multitasking method to infer the key genes for PAAD occurrence [7]. HiFreSP, a high-frequency subpath mining method, was proposed to identify cancer risk factors and the molecular pathways related to prognosis [8].

From the perspective of digital signal processing, S. Wang and X. Wang introduced a two-dimensional wavelet into protein structure prediction for denoising [9]. Lin et al. used stationary wavelet transform for sequence similarity analysis, where wavelet transform was used to convert complex numbers obtained from cluster mapping into feature vectors [10].

Recently, combined with a convolutional neural network (CNN), a stacked ensemble model was introduced to extract features and achieve satisfactory prognosis performance [11]. Chen et al. proposed a deep-learning capsule network, CapsNetMMD, which can transform gene identification into a supervised classification problem, outperforming classic machine learning methods [12]. DeepType is also a deep learning-based algorithm for cancer subtype classification, which effectively combines supervised learning and unsupervised clustering [13]. Hao et al. proposed a sparse deep neural network, PASNet, for prognostic analysis and identification of complex biological processes related to prognostic pathways [14].

Both CNN for feature extraction and discrete wavelet transform in signal processing were adopted to tumor subtype classification in medical imaging [15]. Meintjes et al. utilized CNN and continuous wavelet transform in the classification of heart sounds [16]. In the segment study of brain tumor, wavelet transform can enhance the CNN structure, thus leading to effectively improve the image processing capability [17].

Although massive contributions were completed in cancer diagnosis and treatment, to significantly reduce its morbidity and mortality, it is still required to thoroughly identify feasible biomarkers for the occurrence and development in combination with multisource data and to comprehensively reveal its tumorigenesis mechanisms.

Therefore, this work attempted to identify the combinatorial predictors for PAAD prognosis through the integration of multisource genomic, epigenomic, and clinical information. We proposed a novel deep learning-based method with wavelet feature selection to construct the PAAD prognostic model. With the mRNA profiling (RNA-seq), DNA methylation, and corresponding clinical information retrieved from The Cancer Genome Atlas (TCGA), the constructed model was validated by the predictive ROC (receiver operating characteristics) test on the 3-year and 5-year survival rates, respectively. Together, our proposed model was compared with classic LASSO and previous wavelet-based approaches quantitatively, rendering enhanced performance with less predictor consideration.

In the rest of the paper, Section 2 presents a detailed method proposal and multisource datasets under study; Section 3 discusses the validation and comparison results for the method; Section 4 draws the conclusion and highlights the future direction.

## 2. Materials and Methods

**Figure 1:**
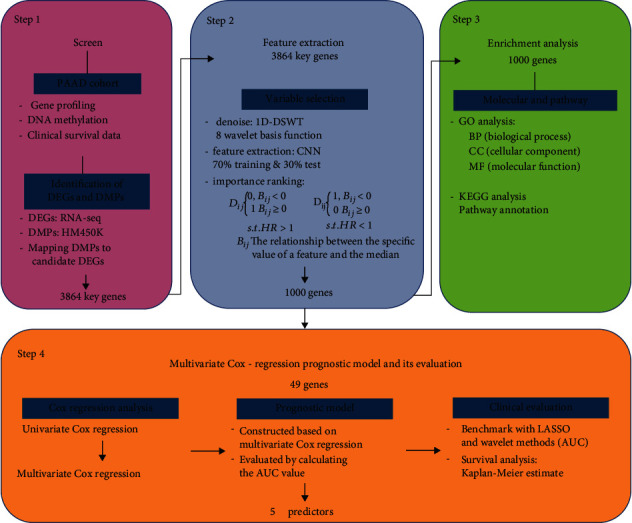
The pipeline to identify key genes and construct a prognostic risk model. The initial three parts mainly cover the procedures to retrieve, identify, and analyze key genes. The last part is the construction of the prognostic risk model.

Figure 1 depicts the analytic pipeline to identify candidate prognostic predictors based on multisource genomic and epigenomic profiling and clinical information for PAAD. The initial step is to retrieve multisource mRNA profiling (RNA-seq) and DNA methylation (Infinium HumanMethylation450K) data from TCGA and determine differentially expressed genes (DEGs) with the cutoff criteria (∣log FC | ≥1.5 and the adjusted *p* value < 0.05) and determine differential methylation probes (DMPs) with the cutoff criteria (∣deltaBeta | >0.2 and the adjusted *p* value < 0.05). The key gene candidates are filtered through integrating the DEGs and DMPs; specifically, the key genes are the DEGs that interact with differential methylation probes. Secondly, a new DSWT method based on deep learning is used for feature extraction from key genes. And functional enrichment analysis was performed on the screened key genes to identify functional clues. Enrichment analysis includes Gene Ontology (GO) and Kyoto Encyclopedia of Genes and Genomics (KEGG) analysis. After performing Cox regression analysis on the genes obtained after feature selection of 56 kinds of wavelet basis functions, a prognostic model of five genes was finally formulated.

Compared with the traditional LASSO method and the latest method SWT-CNN, the predictive ability of the proposed deep learning method is significantly higher than that of LASSO [18] and SWT-CNN [19].

### 2.1. Gene Expression Data and DNA Methylation Data Information

The gene expression profiling, DNA methylation, and clinical information in this study were retrieved from TCGA, including 178 tumor and 4 normal samples.  Table 1 lists the data statistics adopted in this study. We detected the DEGs between tumor and normal tissue samples using edgeR [20], with the ∣log FC | ≥1.5 and adjusted *p* value < 0.05 and identified the DMPs with the ∣deltaBeta | >0.2 and adjusted *p* value < 0.05 as the cutoff criteria using ChAMP [21].

### 2.2. Deep Learning-Based DSWT Method for Feature Selection

#### 2.2.1. One-Dimensional Stationary Wavelet Transform for Noise Removal

Wavelet transform has been widely used in signal processing and computer vision [22, 23]. Regarded as an extension of short-time Fourier transform (STFT), it can effectively solve the limitation that the shape and size of the STFT window do not change with frequency. In many signals, the low-frequency part is particularly important. Based on the Mallet algorithm, discrete wavelet transform (DWT) can effectively extract the low-frequency part of the signal, also known as noise removal [24].

With a mother wavelet *ψ*((*t* − *b*)/*a*), the continuous wavelet transform of a function *x*(*t*) can be formulated as.

(1) $$\begin{array}{c} {CWT}_{x}\left(a,b\right)=\frac{1}{\sqrt{a}}\int_{-\infty }^{\infty }x\left(t\right)\psi \left(\frac{t-b}{a}\right)dt, \end{array}$$

where *a* denotes the scale coefficient and *b* the translation distance [25]. Frequently, the two parameters can be discretized by the 2-base power series as.

(2) $$\begin{array}{c} a=2^{j},b=k2^{j},\;\text{for}\forall j,k\in \mathbb{Z}. \end{array}$$

The mother wavelet *ψ*(*t*) can be further reformulated as

(3) $$\begin{array}{c} \psi_{j,k}\left(t\right)=\frac{1}{\sqrt{2^{j}}}\psi \left(\frac{t}{\sqrt{2^{j}}}-k\right). \end{array}$$

Thus, the corresponding discrete wavelet transform is derived as

(4) $$\begin{array}{c} {DWT}_{x}\left(j,k\right)=\frac{1}{\sqrt{2^{j}}}\int_{-\infty }^{\infty }x\left(t\right)\psi_{jk}\left(\frac{t}{\sqrt{2^{j}}}-k\right)dt, \end{array}$$

where *j* denotes the scale coefficient and *k* is the translation distance, respectively.

**Figure 2:**
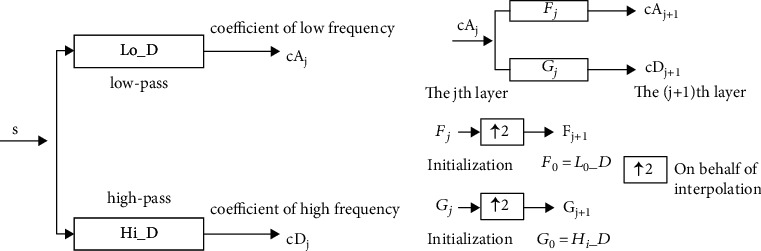
DSWT algorithm. (a) The input signal is divided into two coefficient sets, high and low; (b) the low-frequency coefficient set is decomposed by the method in (a). Lo_D: low pass filter; Hi_D: high pass filter.

In this study, we adopted the one-dimensional discrete stationary wavelet transform (DSWT) due to its translational invariance. Compared with DWT, the length of each channel coefficient after decomposition is equal to the original signal. The DSWT algorithm contains two steps: (a) the input signal is divided into two coefficient sets, high and low; (b) the low-frequency coefficient set is decomposed by the method in (a). The details are depicted in Figure 2.

We use the wavelet to denoise the gene expression profiles. Here, we mainly choose the basis function of DSWT based on three parameters: (a) decomposition layers, (b) the family of wavelets, and (c) the corresponding basis function in a family of wavelets.

The number of decomposition layers has a great influence on noise removal. Too few layers may lead to a poor denoising effect, but too many layers may also lead to signal distortion. Here, we select the optimal number of decomposition layers according to the degree of distortion of the signal when setting different decomposition layers.

Here, we mainly consider 8 wavelet families and corresponding 56 basis functions, which are daubechies (basis function: db*i*, *i* = 2, 3, 4, 5, 6, 7, 8), symlets (basis function: sym*i*, *i* = 2, 3, 4, 5, 6, 7, 8), coiflets (basis function: coif*i*, *i* = 2, 3, 4, 5), biorSplines (basis function: bior*i*, *i* = 1.1, 1.3, 1.5, 2.2, 2.4, 2.6, 2.8, 3.1, 3.3, 3.5, 3.7, 3.9, 4.4, 5.5, 6.8), reverseBior (basis function: rbio*i*, *i* = 1.1, 1.3, 1.5, 2.2, 2.4, 2.6, 2.8, 3.1, 3.3, 3.5, 3.7, 3.9, 4.4, 5.5, 6.8), haar, dmeyer, and Fejer-Korovkin (basis function: fk*i*, *i* = 4, 6, 8, 14, 18, 22), respectively (Table 2).

For its outstanding capabilities in processing matrix-wise information, the CNN method develops rapidly and is applied in quite a few topics [26, 27]. Here, we utilized it in feature extraction. The DSWT results serve as the network input; specifically, 70% of the input samples were selected as the training set and 30% as the test set.

#### 2.2.2. Step Function for Determining Gene Scores

A score metric is assigned to each gene to evaluate its importance in prognosis. There are two procedures to acquire the gene score: (a) the gene weight matrix *C*_*n*×*m*_ obtained by CNN and (b) sparse matrix *D*_*q*×*m*_. Matrix *C* is calculated according to the output matrix *A* of the maximum pooling layer of CNN. *A* is a three-dimensional matrix with dimension *m* × *n* × *p* (*m* is the sample size of the input of CNN, *n* is the number of features, and *p* is the number of channels). After averaging the matrix *A*, according to the number of channels *P*, we get the matrix *C*_*n*×*m*_. We assume the expression matrix of DEGs is *B*_*q*×*m*_. Here, we obtain the matrix *D* by the formula similar to a step function [28].

(5) $$\begin{array}{c} D_{ij}=\left\{\begin{array}{c} 0,\;B_{ij}<0\\ 1\;B_{ij}\geq 0 \end{array}\right.,\text{s}.\text{t}.\;HR>1, \end{array}$$

(6) $$\begin{array}{c} D_{ij}=\left\{\begin{array}{c} 1,\;B_{ij}<0\\ 0\;B_{ij}\geq 0 \end{array}\right.,\text{s}.\text{t}.\;HR<1, \end{array}$$

where *HR* is obtained by univariate Cox analysis of DEGs. The relationship between *B*_*ij*_ and 0 is corresponding to the gene expression level and its median. And the matrix *E*_*q*×*n*_ can be denoted as.

(7) $$\begin{array}{c} E_{q\times n}=D_{q\times m}C_{n\times m}^{T}. \end{array}$$

Then, individual gene score (GS) is estimated as.

(8) $$\begin{array}{c} {GS}_{i}=\frac{1}{n}\overset{n}{\underset{i=1}{\sum }}\overset{n}{\underset{j=1}{\sum }}E_{ij}. \end{array}$$

#### 2.2.3. Functional Enrichment Analyses

Gene Ontology (GO) analysis is utilized to describe three types of gene products, biological process (BP), molecular function (MF), and cellular component (CC). Then, we further systematically interrogated KEGG pathways to link genomic profiling status to higher-order functional information.

### 2.3. Cox Regression Analysis

The Cox Proportional Hazards model, essentially a statistical regression model, is adopted to investigate the association between the survival time of patients and potential predictive factors [29].

#### 2.3.1. Univariate Cox Regression Analysis

The genes obtained by feature selection were analyzed by a univariate Cox regression model, and the relationship between the gene expression level and its corresponding patient survival time was analyzed. The results of univariate Cox regression analysis included regression coefficient (*β*), risk ratio (95% confidence interval), *z*, Wald test results, and *p* value [29].

#### 2.3.2. Multivariate Cox Regression Analysis

Cox risk regression analysis was performed to assess the mutual effect of several predictive factors on survival outcomes. Specifically, initial screening was carried out on the genes obtained by univariate Cox regression; then, multivariate Cox regression analysis was performed on the screened genes. After both univariate and multivariate analyses, a candidate gene with its *p* value less than 0.05 can be considered an independent predictor.

### 2.4. Construction of Multivariate Cox Regression Prognostic Model

The prognostic risk score (PRS) for a multivariate Cox regression model was calculated based on a linear combination of regression coefficients and the corresponding gene expression level, respectively.

(9) $$\begin{array}{c} \text{PRS}=\overset{N}{\underset{i=1}{\sum }}{\text{Exp}}_{i}*\;\beta_{i}. \end{array}$$

The median threshold of total risk scores divides the samples into the high-risk and low-risk groups.

To analyze the relationship between PAAD patients' risk score and overall survival and their risk score, *p* value < 0.05 was the significance test level, and the risk score had a significant impact on the overall survival of PAAD patients. The prognosis was predicted by the Kaplan-Meier curve and the Cox regression model, and the sensitivity was tested by the subject's working curve.

Furthermore, in evaluating model performance, the true positive rate (TPR, or sensitivity), a measure of the proportion of positive cases in the data that are correctly classified, is defined as

(10) $$\begin{array}{c} \text{TPR}=\frac{\text{TP}}{\text{TP}+\text{FN}}, \end{array}$$

where TP denotes true positive and FN for false negative. And the false positive rate (FPR or fall-out), the proportion of negative cases incorrectly classified as positive cases in the data, is introduced as

(11) $$\begin{array}{c} \text{FPR}=\frac{\text{FP}}{\text{FP}+\text{TN}}, \end{array}$$

where FP denotes false positive and TN for true negative. A ROC (receiver operating characteristic) curve, parameterized with the above measures, is utilized to evaluate the performance of a classification model at all classification thresholds. An AUC (area under the ROC curve) is introduced to measure the classification efficiency.

## 3. Results

We retrieved mRNA profiling data (RNA-seq) and filtered the candidate genes satisfying the cutoff criteria, ∣logFC | ≥1.5, and adjusted *p* value < 0.05, as the DEGs; in the DNA methylation dataset (Infinium HumanMethylation450K), we filtered the methylation positions satisfying the cutoff criteria, adjusted *p* value < 0.05, and ∣deltaBeta | >0.2 as DMPs, then identified the key genes related to DMPs using ChAMP [21]. Then, we merged the DEGs and the genes related to DMPs as the key genes. After the screening analysis, totally 3864 gene candidates were obtained.

### 3.1. DSWT-Based Noise Removal Analysis

In this study, through experiment validation, it is most reasonable to set the number of decomposition layers of DSWT at 2. A total of 56 wavelet basis functions are selected to remove the noise. The optimal basis function was obtained after the prognostic analysis. The evaluation criteria of the optimal wavelet basis function include two parts: (a) the prognostic model constructed has the best prognosis performance; (b) the least number of predictors is involved in the corresponding prognostic model. The major performance comparisons are provided in Table 3, and the detailed comparison analyses on the proposed method, classic LASSO, and wavelet-based methods with diverse predictors are listed in additional file 1.

### 3.2. CNN-Based Feature Extraction

**Figure 3:**
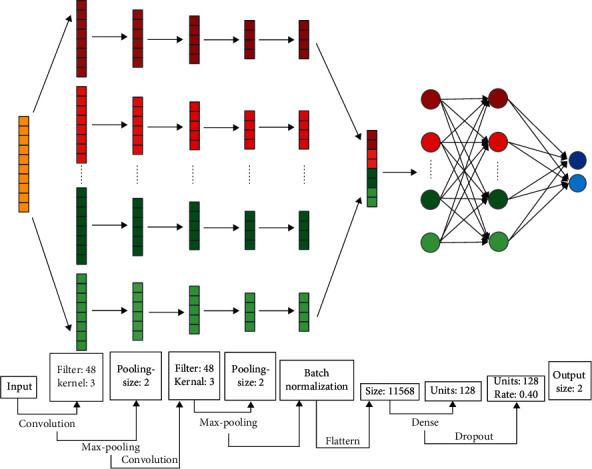
The CNN model structure for feature extraction. The proposed deep model consists of 9 layers, namely, convolutional layer, max-pooling layer, convolutional layer, max-pooling layer, batch normalization layer, flatten layer, hidden layer, dropout layer, and output layer, with the size and link noted above.

Since the gene positions in the expression matrix are not correlated, a one-dimensional CNN is adopted in this study to construct a 9-layer deep neural network, as depicted in Figure 3.

To achieve robust experimental results, the feature extraction process was repeated 100 times. Then, the model with the optimal predicted results of the test dataset was reserved for the subsequent analysis. The step function defined in Section 2.2.2 was utilized to score 3864 key genes, and the functional analysis was based on the top 1000 genes. Then, Cox analyses were performed based on the top 200 genes. According to the performance comparison among the constructed prognosis models, bior1.5 is adopted as the optimal wavelet basis in our proposed model.

### 3.3. Functional Enrichment Analysis

**Figure 4:**
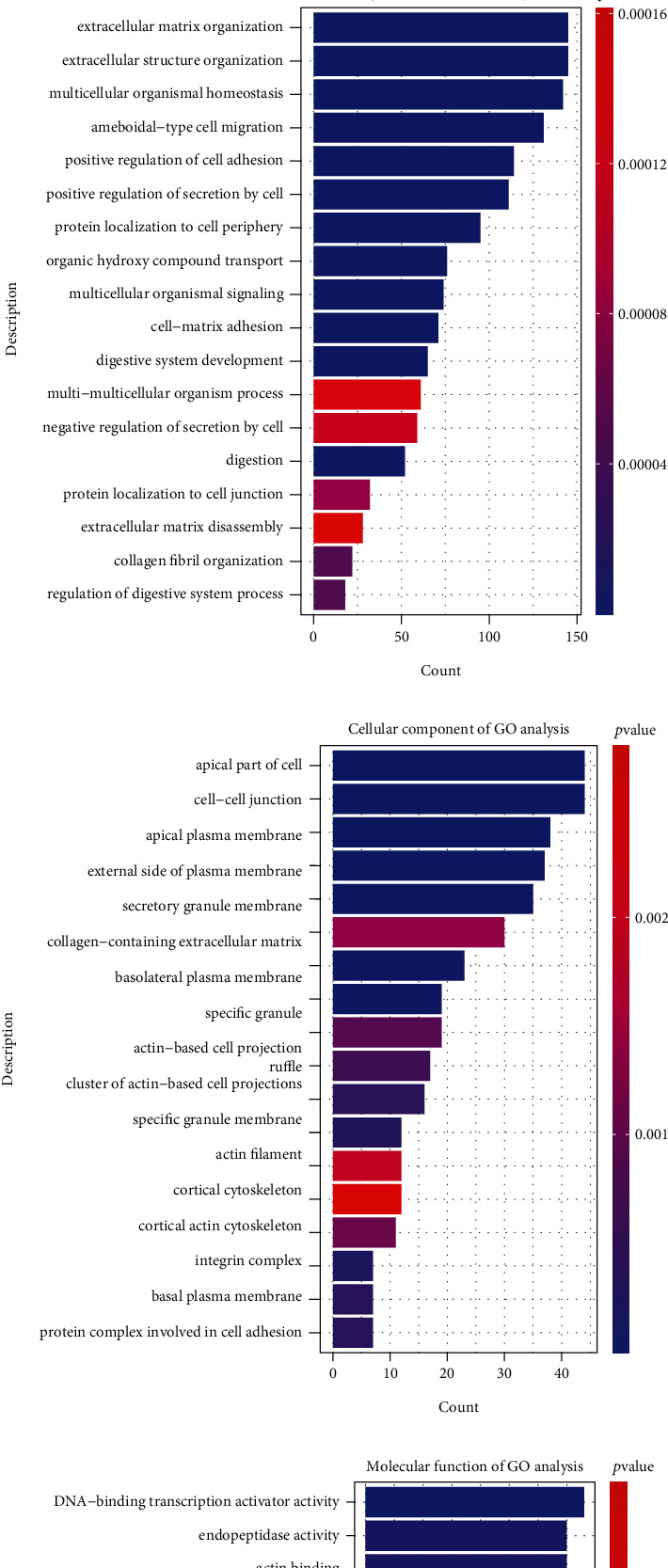
Illustration of KEGG and GO analysis results for the key genes in PAAD. (a) the KEGG pathways of the key genes; (b) the biological process (BP); (c) the cellular component (CC); (d) the molecular function (MF).

The key genes identified from the feature selection were further performed with GO and KEGG analyses, respectively.  Figure 4 depicts the GO function and KEGG pathway enrichment results for the key genes.

The significantly enriched KEGG pathways of the DEGs are illustrated in Figure 4(a). These pathways mainly include MAPK signaling pathway, pancreatic cancer, pancreatic secretion, and ECM-receptor interaction. The full results of KEGG pathway analyses are shown in additional file 2. In Figures 4(b)–4(d), the results indicate that the key genes were mainly enriched in these BP terms as extracellular structure organization, extracellular matrix organization, digestion, and cell adhesion; and CC terms cover secretory granule membrane, cell-cell junction, and actin filament; MF terms include serine-type peptidase activity, endopeptidase activity, and actin binding. All the results of GO analysis about the BP, CC, and MF terms are shown in additional files 3 to 5, respectively.

### 3.4. Cox Regression Analysis and Construction of the Prognostic Model

First, univariate Cox regression analysis was carried out on key genes to filter satisfactory candidate predictors, wherein the stepwise regression was conducted on the results of the univariate Cox regression analysis. Afterwards, the prognostic models are further constructed with multivariate Cox analysis based on the initially filtered candidates.

The prognostic ability of the established model was evaluated by calculating the AUC value of the ROC curve to select the optimized prognostic model. The detailed decomposition results are depicted in Table 3. According to Table 3, the optimal prognosis model was acquired when the biorSplines wavelet family was selected and the smoothness was 1.5.

Comparatively, the prognostic model with the biorSplines basis bior1.5 can achieve the AUC value 0.937 with 5 predictors in the 5-year survival case; while in the 3-year survival case, the reverseBior wavelet rbio2.8 can achieve the AUC value of 0.852 on 10 predictors. However, the classic LASSO method requires at least 11 predictors to achieve the AUC, 0.782 for the 3-year case, and 0.802 for the 5-year case; the previous SWT-CNN requires at least 8 genes to achieve the AUC 0.721 for the 3-years and 0.859 for the 5-year case.

The results of our proposed method outperformed the other two methods overall; thus, the biorSplines basis was adopted as the variable selection in constructing the prognosis model. Then, univariate and multivariate Cox regression analyses were further performed on the predictor candidates, detailed in Table 4.

Table 4 depicts the univariate and multivariate Cox regression analysis to filter the prognosis risk candidates, wherein five molecules, namely, MCF2L, FAM184B, KRT19, GBP4, and GALNT5, were determined as the predictors. Thus, after the above quantitative comparison, the final optimal prognosis model is denoted with the five molecular risk predictors.

(12) $$\begin{array}{c} \text{PRS}=\left(-0.342*{\text{Exp}}_{\text{FAM}184\text{B}}\right)+\left(0.300*{\text{Exp}}_{\text{KRT}19}\right)+\left(0.318*{\text{Exp}}_{\text{GBP}4}\right)+\left(-0.070*\text{Ex}{\text{p}}_{\text{GALNT}5}\right)+\left(-0.412*{\text{Exp}}_{\text{MCF}2\text{L}}\right). \end{array}$$

### 3.5. Kaplan-Meier Survival Analysis

Each patient's risk score was calculated using the prediction function based on the constructed prognosis model in Equation (12). The cohorts were divided into two groups, the high-risk group and the low-risk group, respectively.

**Figure 5:**
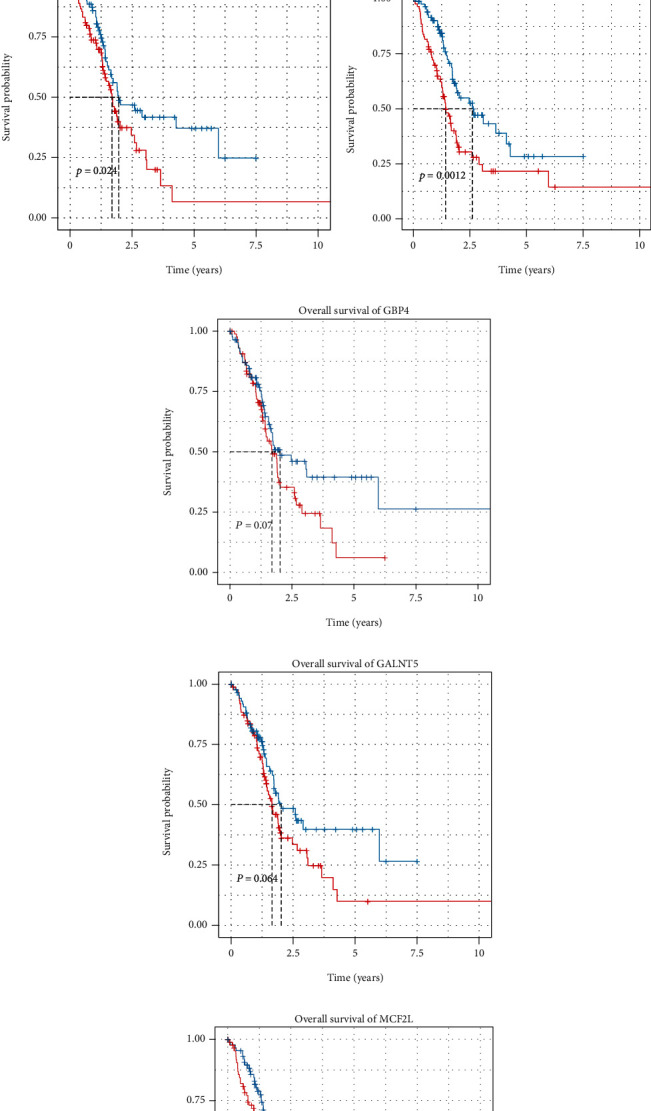
Kaplan-Meier survival analysis of the 5-molecule signature and individual predictors. (a) Kaplan-Meier survival curve analysis for overall survival of PAAD patients using the 5-molecule signature. Survival in the low-risk group was much longer than in the high-risk group; (b) overall survival distribution of FAM184B; (c) overall survival distribution of KRT19; (d) overall survival distribution of GBP4; (e) overall survival distribution of GALNT5; (f) overall survival distribution of MCF2L.

The survival rates were calculated from the high-low risk groups, and the corresponding Kaplan-Meier survival diagram is illustrated in Figure 5(a); and for the comparison of prognosis potentials of individual molecules, the other five molecular predictors are provided in Figures 5(b)–5(f). Noticeably, the three predictors involved in the 5-molecule prognosis model are statistically significant, namely, FAM184B, KRT19, and MCF2L (log-rank test *p* value < 0.05). Noticeably, although the five protein-coding genes have not been reported in PAAD, they have interfered with other disease or pathway dysfunction, for example, interferon-gamma signaling and cytokine signaling in immune system, GPCR pathway, and p75 NTR receptor-mediated signaling.

### 3.6. Comparison with Other Typical Methods

**Figure 6:**
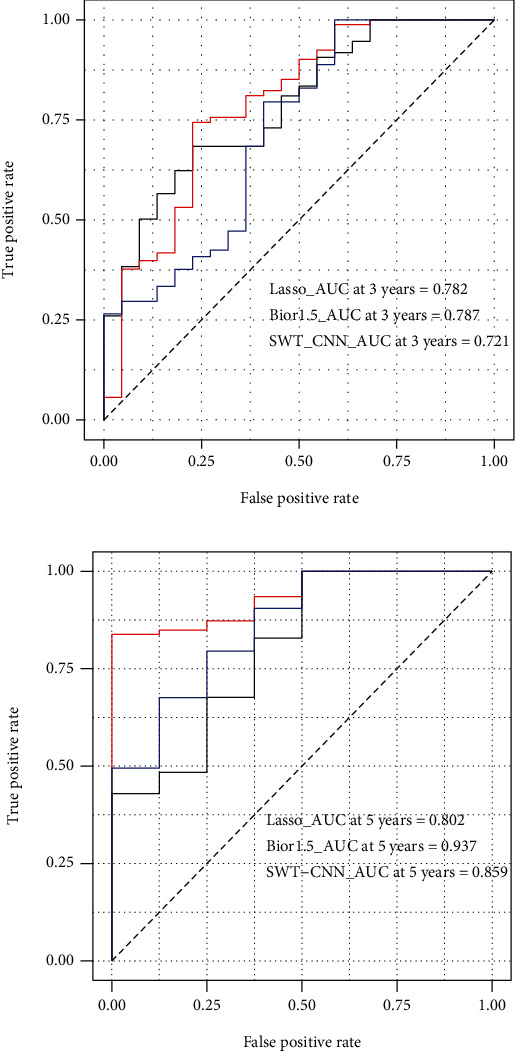
Performance comparison with the SWT-CNN and traditional LASSO methods. (a) AUC at 3 years: bior1.5_AUC at 3 years = 0.787, lasso_AUC at 3 years = 0.782, and SWT-CNN at 3 years = 0.721; (b) AUC at 5 years: bior1.5_AUC at 5 years = 0.937, lasso_AUC at 5 years = 0.802, and SWT-CNN at 5 years = 0.859.

The prognostic power of the established model was evaluated by the AUC value of the ROC curve with the SWT-CNN and classic LASSO methods (Figure 6), where the AUC measure for the 3- and 5-year terms was compared, respectively. The AUC at 3 years of our proposed model is 0.787, and the AUC at 5 years is 0.937, while for the classic LASSO method, the AUC at 3 years and 5 years are 0.782 and 0.802, respectively. For the SWT-CNN method, the AUC at 3 years and 5 years are 0.721 and 0.859, respectively. The results indicate that the prognostic model proposed had a better sensitivity in predicting the survival risk of PAAD patients.

## 4. Conclusions and Discussion

Pancreatic cancer is a high-incidence tumor type with poor clinical prognosis. There is an urgent need to develop effective methods for the prognosis prediction of pancreatic cancer. Thus, we proposed a novel CNN-based model to enhance prognosis performance in PAAD through combining wavelet transform features. In the work, we integrated mRNA expression and DNA methylation, together with cohort clinical information into PAAD prognosis formulation, from an ensemble perspective of multiple omics data.

Firstly, with the noise reduction functionality of wavelet transform in signal processing, a 1D-DSWT was utilized to denoise the identified key genes, specifically differentially expressed genes overlapping with differentially methylated loci. The number of decomposition layers, wavelet family, and basis function types are considered in the wavelet function selection for denoising, and the low-frequency part of the signal value is reserved for the subsequent analyses.

Then, a 9-layer CNN structure is trained for feature extraction after the above noise reduction process. The weights of candidate genes are obtained from feature extraction, specifically the output matrix of the CNN pooling layer, for subsequent analysis. A scoring measure is proposed for weighting important molecular predictors involved in pancreatic cancer prognosis. The score consists of three parts, the HR value obtained by univariate Cox regression analysis of the key genes, the sparse matrix constructed by the step function of HR value, and the weights derived from the CNN structure.

Through enrichment analysis of the screened genes, it indicates that these identified genes are involved in many biological activities closely related to pancreatic cancer, which manifests the biological significance of the candidates to the prognosis of pancreatic cancer.

The initial candidate genes in the scoring process were adopted for univariate Cox analysis to compress the candidate space; then after multivariate Cox regression, five molecule predictors were screened out to construct the prognosis model. To verify the survival predictive capability, the constructed model and individual predictors have been examined with survival analysis via Kaplan-Meier estimate, respectively. The constructed model has significant statistical predictive ability on high- and low-risk cases (*p* value: 0.00007). For the other three individual factors, it also manifested the underlying survival prognosis significance (*p* value: 0.0012~0.024).

Moreover, to validate the prediction performance and measure the diagnostic accuracy of quantitative tests, the proposed prognosis model was compared with the traditional LASSO and wavelet-based methods in the 3- and 5-year prediction AUC scores, respectively. In three prediction cases, our proposed model with discrete stationary wavelet base (bior1.5) got the optimal results, especially in the 5-year case. We applied the proposed method to the other tumor type, stomach adenocarcinoma (STAD). The results were superior to some previous methods, proving that our proposed method has a certain degree of robustness. The data and specific results are shown in additional file 6.

In the future study, the proposed method will be applied to diverse cancer types and multiple clinical and biological profiling data to further test its robustness and flexibility in tumor prognosis. Furthermore, combined with the characteristics of pancreatic cancer data distribution, specific processing measures for high-dimensional unbalanced data are another focus in the subsequent analysis. The processing of class imbalanced data can be further combined with feature selection to improve the model accuracy and reduce the complexity of the model.

## Figures and Tables

**Table 1:** Statistics of gene expression profiling, DNA methylation, and clinical cohort information of PAAD under study.

PAAD clinical cohort	Statistics
Tumor	178
Normal	4
Histological type	
PDAC	146
Pancreas-colloid carcinoma	4
Other	28
Survival status (tumor)	
Living	86
Deceased	93
Follow-up (months)	0.13-132.97
Age (years)	
Range	35-88
Median	61.3
Gender (tumor)	
Male	98
Female	80
#. (Epi)genomics data	
mRNA profiling (DEGs)	17240
DNA methylation (DMPs)	10648

**Table 2:** A total of eight typical wavelet family functions adopted in the study.

Wavelets	Description
daubechies	*ψ*(*t*) = ∑_*k*_*g*_*k*_*ϕ*(2*t* − *k*), *ϕ*(*t*): scale function, *g*_*k*_ : weight
symlets	An improvement over db and approximate symmetric wavelet function
coiflets	Compared with db, *ψ*(*t*) and *ϕ*(*t*) have better symmetry
biorSplines	The input signal *f*(*t*), $\;\left\{\begin{array}{c} \text{Decompos}:C_{j,k}\left(t\right)=\int f\left(t\right){\tilde{\psi }}_{j,k}\left(t\right)dt\\ \text{Reconstruct}:f\left(t\right)=\sum_{j,k}{\tilde{C}}_{j,k}\psi_{j,k}\left(t\right)\\ \text{s}.\text{t}.\;\text{the dual wavelet }\psi_{j,k}\left(t\right)\text{ and }{\tilde{\psi }}_{j,k}\left(t\right) \end{array}\right.$
reverseBior	Biorthogonal spline wavelets for which symmetry and exact reconstruction are possible
haar	$\psi \left(t\right)=\left\{\begin{array}{c} 1,\left(0\leq t<1/2\right),\\ -1,\left(1/2\leq t\leq 1\right),\\ 0,\text{otherwise} \end{array}\right.$ , $\phi \left(t\right)=\left\{\begin{array}{c} 1,\left(0\leq t\leq 1\right),\\ 0,\text{othewise} \end{array}\right.$
dmeyer	Discrete Meyer wavelet, FIR-based approximation of Meyer wavelet
Fejer-Korovkin	It has an optimal progressive resolution.

**Table 3:** Performance comparison among the eight typical wavelet-based and classic LASSO and SWT-CNN methods.

Method	Basis	AUC at 3 years	AUC at 5 years	Predictors
haar	haar	0.618	0.756	5
dmeyer	dmeyer	0.823	0.869	7
symlets	sym3	0.806	0.904	5
coiflets	coif4	0.696	0.788	5
daubechies	db7	0.804	0.867	5
biorSplines	bior1.5	0.787	0.937	5
reverseBior	rbior2.8	0.852	0.897	10
Fejer-Korovkin	fk14	0.672	0.861	5
SWT-CNN	sym4	0.721	0.859	8
LASSO		0.782	0.802	11

**Table 4:** The univariate and multivariate Cox regression analysis results.

	Univariate Cox		Multivariate Cox	
Candidates	HR (95% CI)	*p* value	HR (95% CI)	*p* value

ABHD8	0.5712 (0.4411-0.7397)	0.00001		
MCF2L	0.58195 (0.456-0.7426)	0.00003	0.6378 (0.454-0.897)	0.0097
FAM184B	0.6376 (0.5084-0.7997)	0.00010	0.7063 (0.517-0.965)	0.0287
GPRC5A	1.27261 (1.1239-1.441)	0.00010	1.0309 (0.804-1.323)	0.8108
KRT19	1.4003 (1.1809-1.6606)	0.00010	1.3305 (1.005-1.761)	0.0460
PADI1	1.1260 (1.0585-1.1978)	0.00020		
TNS4	1.1852 (1.0839-1.2961)	0.00020		
SEMA3C	1.29315 (1.1238-1.488)	0.00030		
MUCL3	1.0985 (1.0426-1.1572)	0.00040		
RNF223	1.24345 (1.102-1.4031)	0.00040		
PCAT2	1.2261 (1.0947-1.3733)	0.00040		
C6orf132	1.3850 (1.1484-1.6703)	0.00070		
C5orf49	0.8046 (0.7097-0.9123)	0.00070		
GABBR1	0.73285 (0.6096-0.881)	0.00090		
ARID3A	0.6824 (0.5442-0.8558)	0.00090		
FOSL1	1.2481 (1.0938-1.4242)	0.00100		
CHGA	0.9029 (0.8494-0.9598)	0.00100		
KRT8	1.4131 (1.1458-1.7428)	0.00120		
PLS1	1.30127 (1.1046-1.533)	0.00160		
CNIH2	0.8180 (0.7215-0.9274)	0.00170		
RYK	1.6625 (1.2059-2.2921)	0.00190		
CTSE	1.1388 (1.0482-1.2372)	0.00210		
GBP4	1.2933 (1.0965-1.5255)	0.00230	1.3814 (1.139-1.676)	0.0010
GALNT5	1.1870 (1.0626-1.3259)	0.00240	0.9135 (0.743-1.123)	0.0391
MAPT	0.83235 (0.739-0.9375)	0.00250		
TRIM59	1.4513 (1.1282-1.8671)	0.00370		
PLAC8	1.2037 (1.0616-1.3649)	0.00380		
TECPR1	0.6379 (0.4698-0.8663)	0.00400		
GRIN2C	0.7433 (0.6068-0.9105)	0.00420		
RASAL1	1.25077 (1.073-1.4579)	0.00420		
CALHM31	1.1261 (1.0353-1.2249)	0.00560		
FGF12	0.8187 (0.7081-0.9467)	0.00690		
TCEA2	0.7593 (0.6204-0.9293)	0.00760		
KRT5	1.0792 (1.0202-1.1416)	0.00790		
TINAG1	1.1122 (1.0281-1.2031)	0.00800		
KCNK3	0.8803 (0.8010-0.9674)	0.00810		
CAPN5	1.2323 (1.0548-1.4396)	0.00850		
SLCO4A1	1.1957 (1.0465-1.3663)	0.00860		
KCNK12	0.8240 (0.7122-0.9532)	0.00920		
FSTL4	0.8815 (0.8015-0.9694)	0.00930		
GPR78	1.2335 (1.0527-1.4452)	0.00940		
FOXA2	0.8482 (0.7381-0.9746)	0.02020	1.0812 (0.891-1.313)	0.4300
DUOX2	1.0933 (1.0138-1.1791)	0.02060		
HDAC4	0.7096 (0.5308-0.9487)	0.02060		
MPPED2	0.8509 (0.7413-0.9768)	0.02180		
SDK1	0.8594 (0.7549-0.9784)	0.02200		
STAC	1.1520 (1.0199-1.3012)	0.02280		
VRK2	1.3697 (1.0399-1.8043)	0.02520		
ITGBL1	1.1277 (1.0126-1.2557)	0.02860		

## Data Availability

The datasets used in this study are publicly available in The Cancer Genome Atlas resource portal (Materials and Methods). Additional files 1 to 6 are available at https://github.com/gladex/WavePrognosis.

## Supplementary Materials

Supplementary Materials Additional file 1: the performance comparison of the proposed method. Additional file 2: the detailed results of KEGG pathway analyses. Additional file 3: the detailed results of GO analysis on the BP term. Additional file 4: the detailed results of GO analysis on the CC term. Additional file 5: the detailed results of GO analysis on the MF term. Additional file 6: the validation of the proposed method on the STAD dataset.
