# Supplementary material for: A Wavelet-Based Learning Model Enhances Molecular Prognosis in Pancreatic Adenocarcinoma
Source: Biomed Res Int. 2021 Oct 16;2021:7865856. doi: 10.1155/2021/7865856 (PMC8541860; doi:10.1155/2021/7865856)
Supplement: Supplementary Materials — Additional file 1: the performance comparison of the proposed method. Additional file 2: the detailed results of KEGG pathway analyses. Additional file 3: the detailed results of GO analysis on the BP term. Additional file 4: the detailed results of GO analysis on the CC term. Additional file 5: the detailed results of GO analysis on the MF term. Additional file 6: the validation of the proposed method on the STAD dataset. [file 7865856.f1.zip › Additional file 1 (2).pdf]

Additional file 1

Table 1. The comparison results of PAAD dataset on the proposed method, classic LASSO and previous wavelet-based methods with diverse predictors

	Wavelet function	AUC at 3 years	AUC at 5 years	gene number
	db2	0.707	0.825	7
	db3	0.707	0.825	7
	db4	0.707	0.825	7
**db**	db5	0.787	0.845	6
	db6	0.799	0.866	6
	db7	0.804	0.867	5
	db8	0.707	0.825	5
	bior1.1	0.797	0.858	5
	bior1.3	0.769	0.838	6
	bior1.5	0.787	0.937	5
	bior2.2	0.681	0.809	4
	bior2.4	0.736	0.857	8
	bior2.6	0.707	0.790	6
	bior2.8	0.729	0.724	8
**bior**	bior3.1	0.761	0.908	7
	bior3.3	0.760	0.908	7
	bior3.5	0.760	0.890	6
	bior3.7	0.691	0.800	5
	bior3.9	0.732	0.857	6
	bior4.4	0.751	0.839	4
	bior5.5	0.795	0.881	8
	bior6.8	0.697	0.792	6
	sym2	0.749	0.873	8
	sym3	0.806	0.904	5
	sym4	0.691	0.756	7
**sym**	sym5	0.767	0.855	5
	sym6	0.658	0.837	5
	sym7	0.788	0.825	6
	sym8	0.787	0.845	7
	coif2	0.700	0.785	9
	coif3	0.775	0.745	6
**coif**	coif4	0.696	0.788	5
	coif5	0.700	0.785	9
**haar**		0.618	0.756	5
**dmeyer**		0.823	0.869	7
	rbio1.1	0.759	0.826	11
	rbio1.3	0.726	0.734	5

	rbio1.5	0.766	0.827	8
	rbio2.2	0.823	0.884	7
	rbio2.4	0.799	0.894	9
	rbio2.6	0.790	0.894	9
	rbio2.8	0.852	0.897	10
**rbio**	rbio3.1	0.812	0.834	9
	rbio3.3	0.595	0.719	6
	rbio3.5	0.749	0.774	7
	rbio3.7	0.783	0.858	8
	rbio3.9	0.693	0.725	6
	rbio4.4	0.795	0.824	7
	rbio5.5	0.762	0.760	10
	rbio6.8	0.784	0.817	7
	fk4	0.687	0.873	6
	fk6	0.681	0.859	7
**fk**	fk8	0.682	0.827	4
	fk14	0.672	0.861	5
	fk18	0.689	0.792	7
	fk22	0.733	0.856	8
**SWT-CNN**	sym4	0.721	0.859	8
**LASSO**		0.782	0.802	11

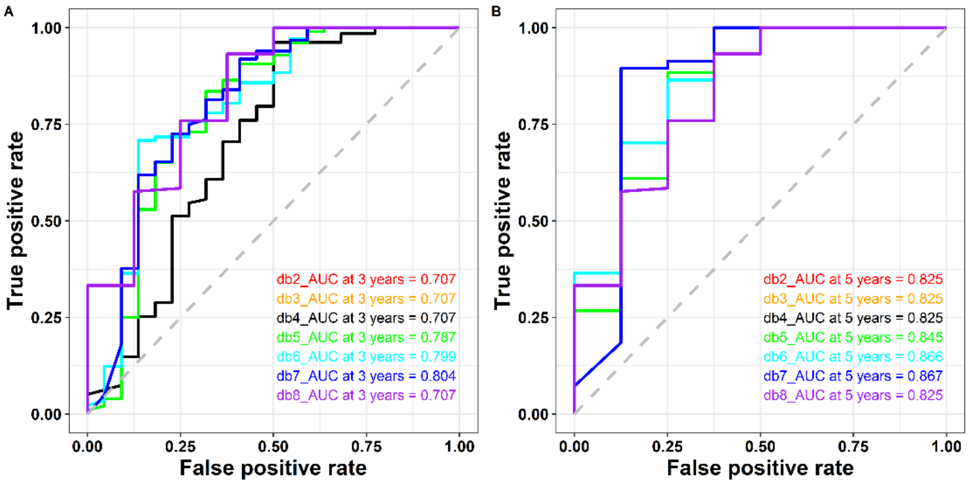

Figure 1. The performance of PAAD dataset on db basis function.

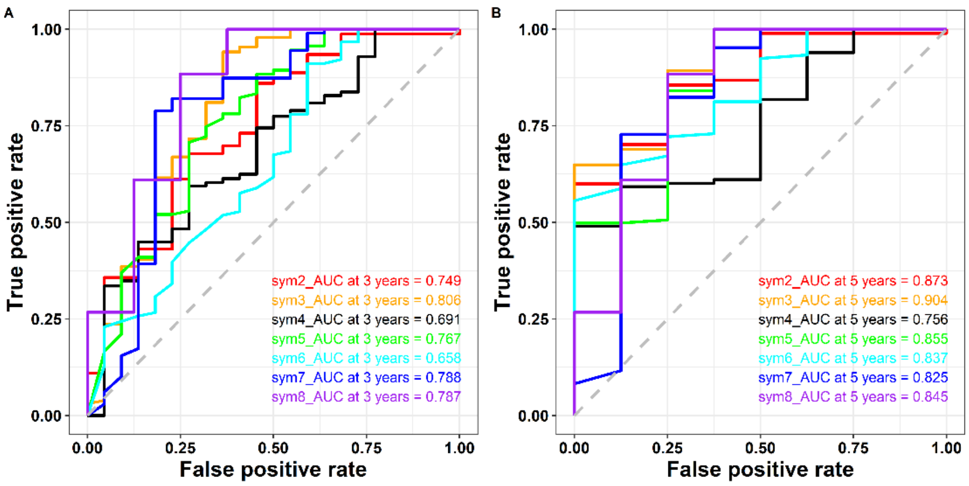

Figure 2. The performance of PAAD dataset on sym basis function.

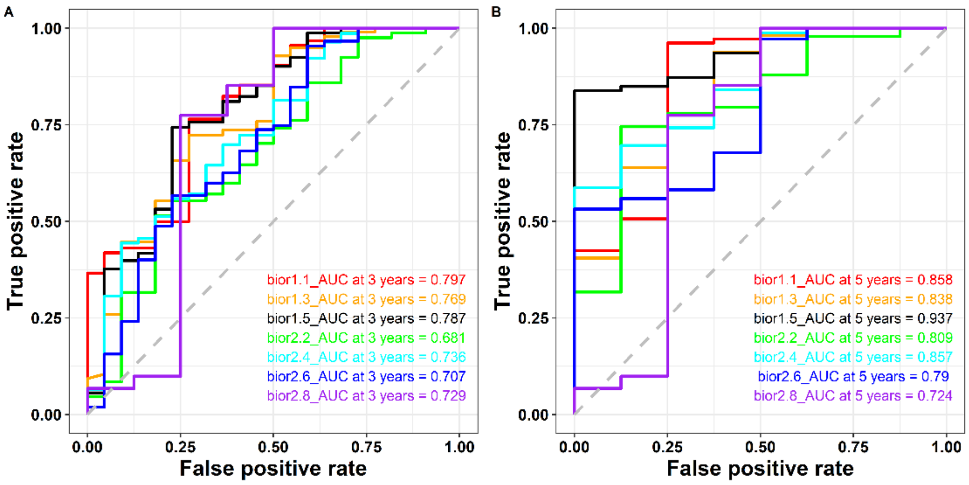

Figure 3. The performance of PAAD dataset on bior basis function (bior1.1~bior2.8).

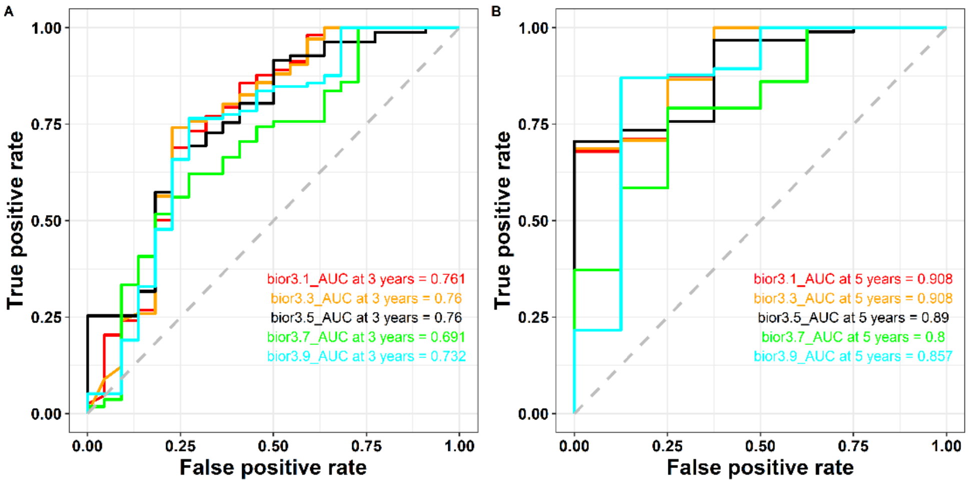

Figure 4. The performance of PAAD dataset on bior basis function (bior3.1~bior3.9).

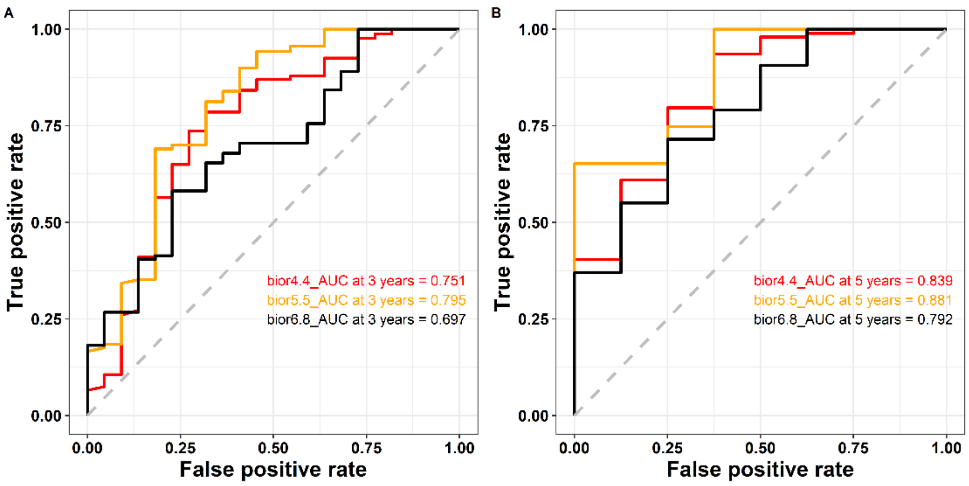

Figure 5. The performance of PAAD dataset on bior basis function (bior4.4~bior6.8).

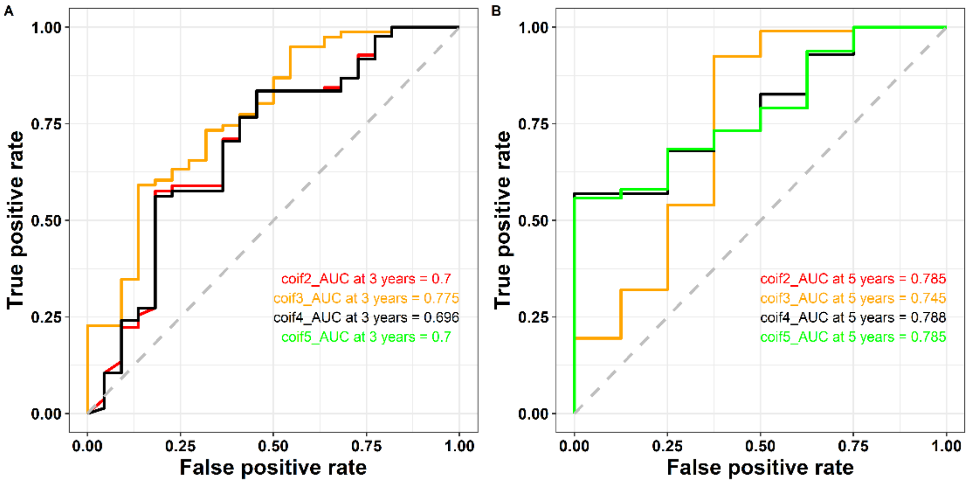

Figure 6. The performance of PAAD dataset on coif basis function.

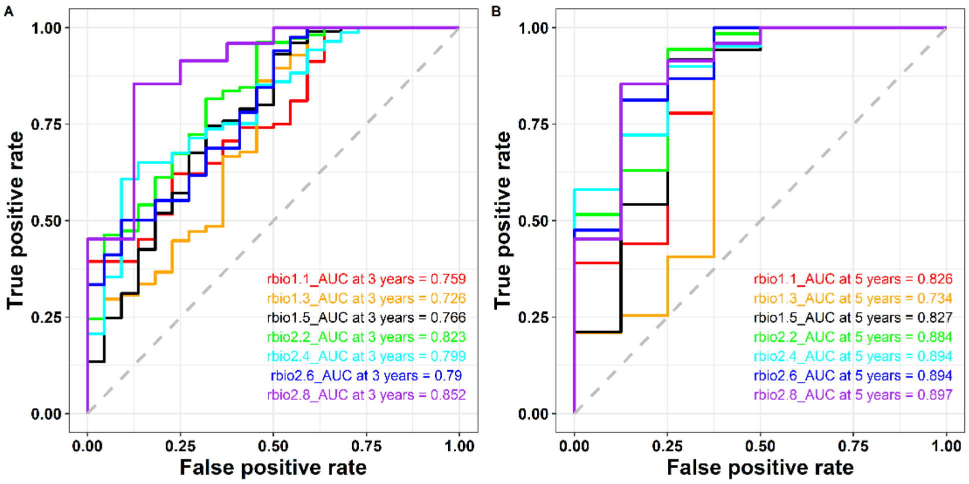

Figure 7. The performance of PAAD dataset on rbio basis function (rbio1.1~rbio2.8).

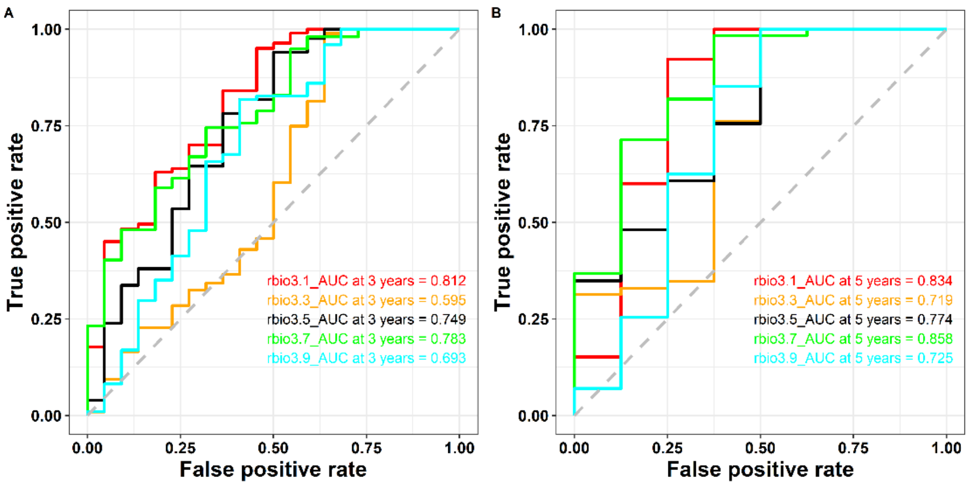

Figure 8. The performance of PAAD dataset on rbio basis function (rbio3.1~rbio3.9).

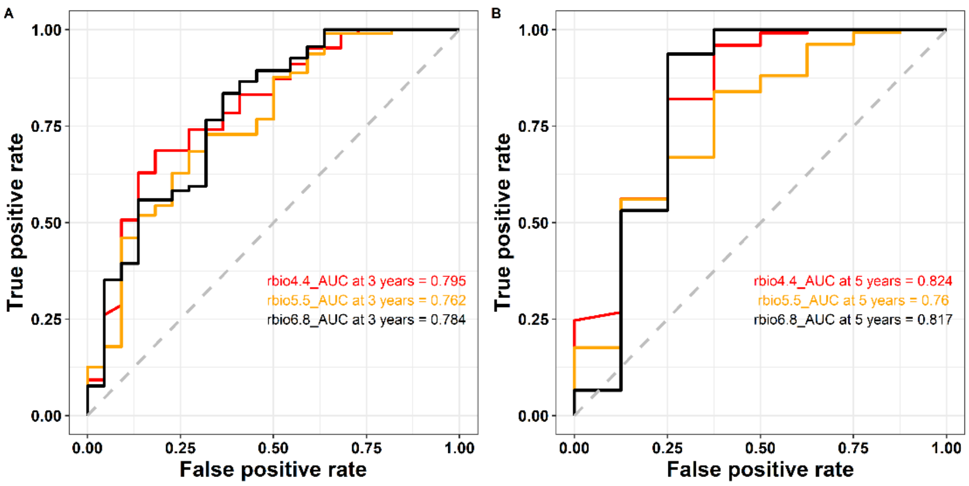

Figure 9. The performance of PAAD dataset on rbio basis function (rbio4.4~rbio6.8).

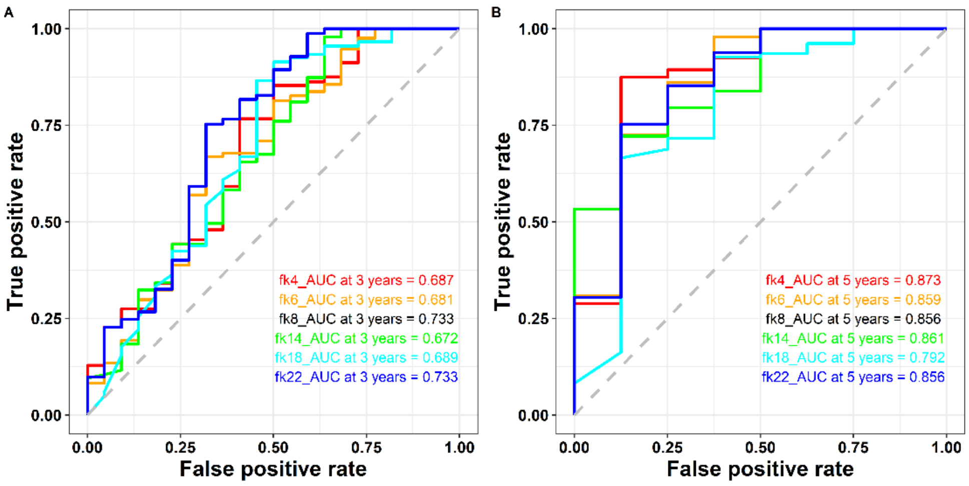

Figure 10. The performance of PAAD dataset on fk basis function.

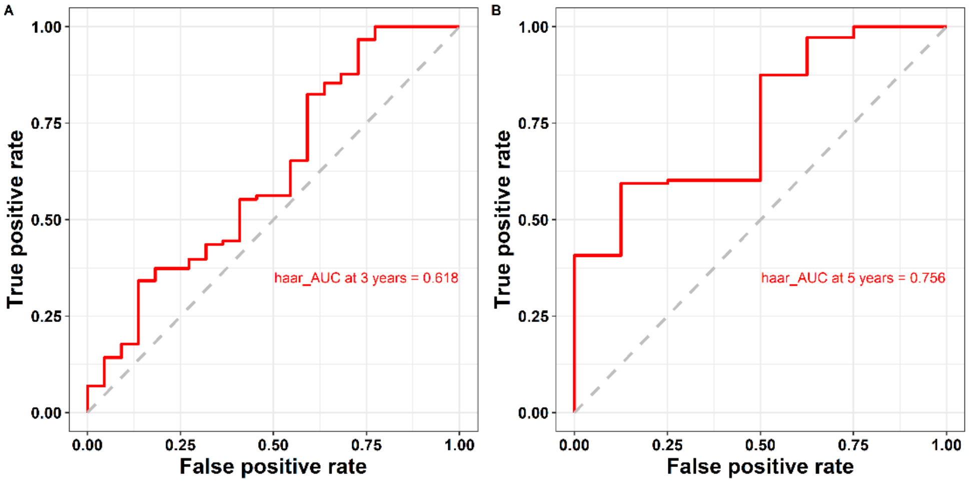

Figure 11. The performance of PAAD dataset on haar basis function.

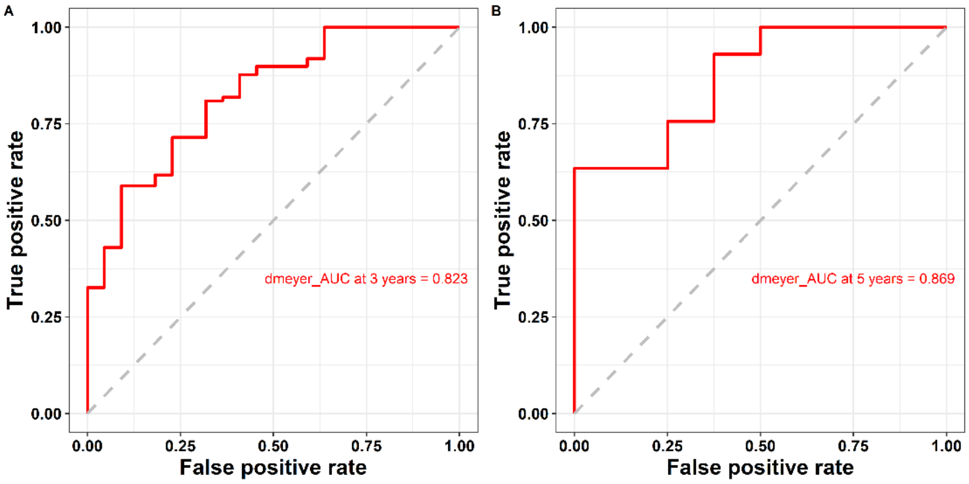

Figure 12. The performance of PAAD dataset on dmeyer basis function.

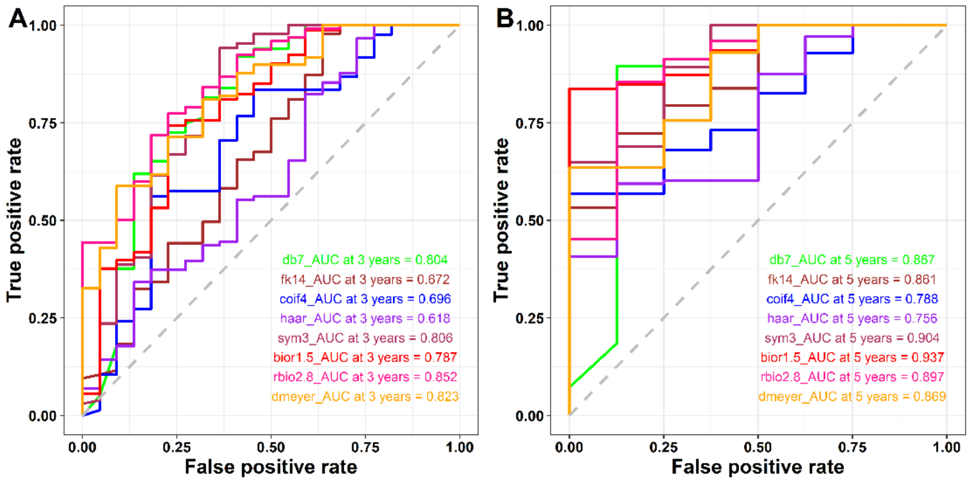

Figure 13. The performance of PAAD dataset on all basis functions (Take the smoothness corresponding to the best result for each basis function).
