# Supplementary material for: A Wavelet-Based Learning Model Enhances Molecular Prognosis in Pancreatic Adenocarcinoma
Source: Biomed Res Int. 2021 Oct 16;2021:7865856. doi: 10.1155/2021/7865856 (PMC8541860; doi:10.1155/2021/7865856)
Supplement: Supplementary Materials — Additional file 1: the performance comparison of the proposed method. Additional file 2: the detailed results of KEGG pathway analyses. Additional file 3: the detailed results of GO analysis on the BP term. Additional file 4: the detailed results of GO analysis on the CC term. Additional file 5: the detailed results of GO analysis on the MF term. Additional file 6: the validation of the proposed method on the STAD dataset. [file 7865856.f1.zip › Additional file 6 (1).pdf]

Additional file 6

1. STAD data

Table 1. Statistics of gene expression profiling, DNA methylation and clinical cohort information of STAD under study.

Item	Statistics
**#. STAD clinical cohort**	
Tumor	375
Normal	32
**Survival status (tumor)**	
Living	204
Deceased	171
**Race (tumor)**	
Asian	74
Black	11
White	238
Not reported	52
**Tumor stage**	
I	53
II	111
III	150
IV	38
Not reported	23
**Follow-up (months)**	0.03-125.8
**Age (years)**	
Range	35-90
Median	63
**Gender (tumor)**	
Male	241
Female	134
**#. (Epi) genomic data**	
mRNA profiling (DEGs)	16486
DNA methylation (DMPs)	6197

2. The results of our proposed methods

Table 1. The detailed analysis results of STAD dataset about comparisons on the adopted wavelet-based, SWT-CNN methods and classic LASSO methods with diverse predictors

	**Wavelet function**	**AUC at 3 years**	**AUC at 5 years**	**gene number**
	db2	0.726	0.725	8
	db3	0.719	0.684	7
	db4	0.712	0.691	5
**db**	db5	0.697	0.720	7
	db6	0.732	0.717	7
	db7	0.670	0.645	4
	db8	0.697	0.720	7
	bior1.1	0.656	0.655	7
	bior1.3	0.725	0.718	6
	bior1.5	0.710	0.706	6
	bior2.2	0.725	0.718	6
	bior2.4	0.707	0.690	6
	bior2.6	0.700	0.719	7
	bior2.8	0.725	0.718	6
**bior**	bior3.1	0.698	0.714	5
	bior3.3	0.701	0.697	5
	bior3.5	0.701	0.697	5
	bior3.7	0.700	0.719	7
	bior3.9	0.714	0.682	7
	bior4.4	0.710	0.706	6
	bior5.5	0.725	0.718	6
	bior6.8	0.710	0.706	6
	sym2	0.726	0.725	8
	sym3	0.719	0.684	7
	sym4	0.726	0.729	5
**sym**	sym5	0.701	0.780	7
	sym6	0.709	0.705	5
	sym7	0.756	0.730	12
	sym8	0.716	0.718	8
	coif2	0.709	0.705	5
	coif3	0.709	0.705	5
**coif**	coif4	0.712	0.691	5
	coif5	0.712	0.691	5
**haar**		0.692	0.681	9
**dmeyer**		0.710	0.706	6
	rbio1.1	0.672	0.771	5
	rbio1.3	0.685	0.666	6

	rbio1.5	0.670	0.623	7
	rbio2.2	0.673	0.695	8
	rbio2.4	0.670	0.623	7
	rbio2.6	0.688	0.636	5
	rbio2.8	0.708	0.648	8
**rbio**	rbio3.1	0.656	0.655	6
	rbio3.3	0.656	0.655	6
	rbio3.5	0.673	0.695	8
	rbio3.7	0.655	0.623	4
	rbio3.9	0.673	0.695	8
	rbio4.4	0.710	0.706	6
	rbio5.5	0.700	0.719	7
	rbio6.8	0.710	0.706	6
	fk4	0.709	0.703	6
	fk6	0.712	0.691	5
**fk**	fk8	0.716	0.718	8
	fk14	0.715	0.705	7
	fk18	0.697	0.720	7
	fk22	0.714	0.693	11
**SWT-CNN**		0.673	0.742	7
**LASSO**		0.682	0.709	14

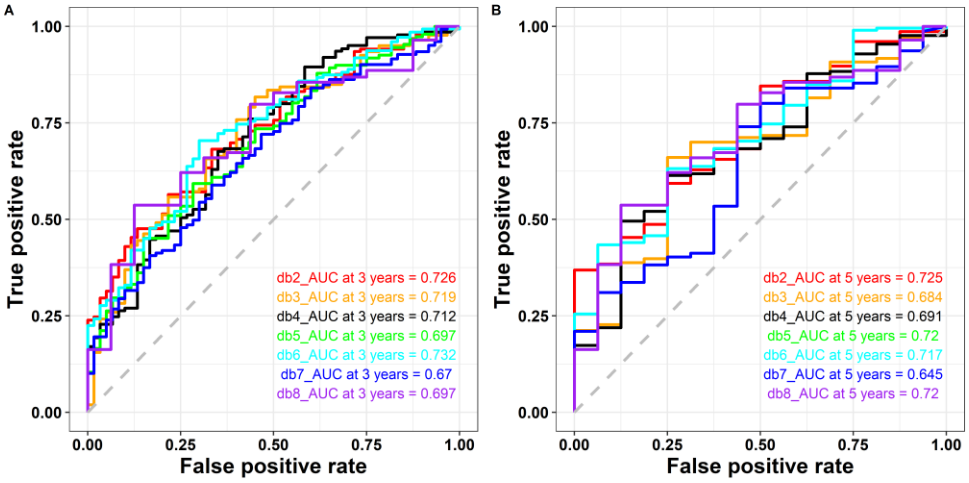

Figure 1. The performance of STAD dataset on db basis function.

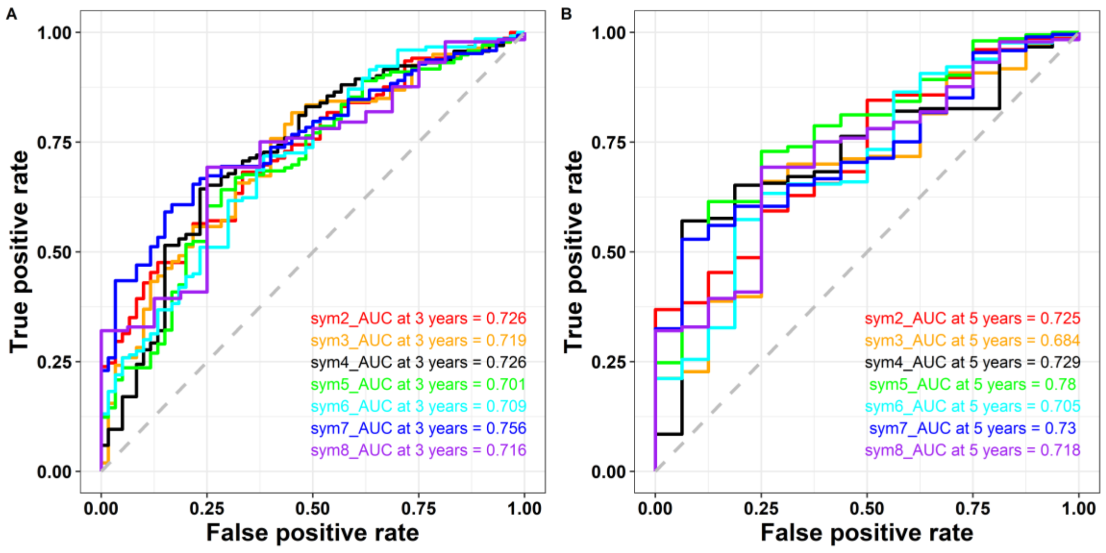

Figure 2. The performance of STAD dataset sym basis function.

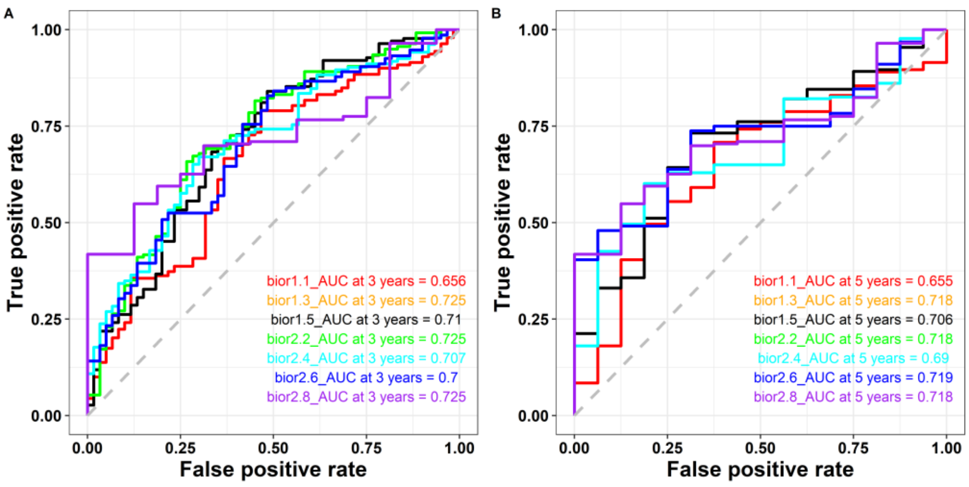

Figure 3. The performance of STAD dataset on bior basis function (bior1.1~bior2.8).

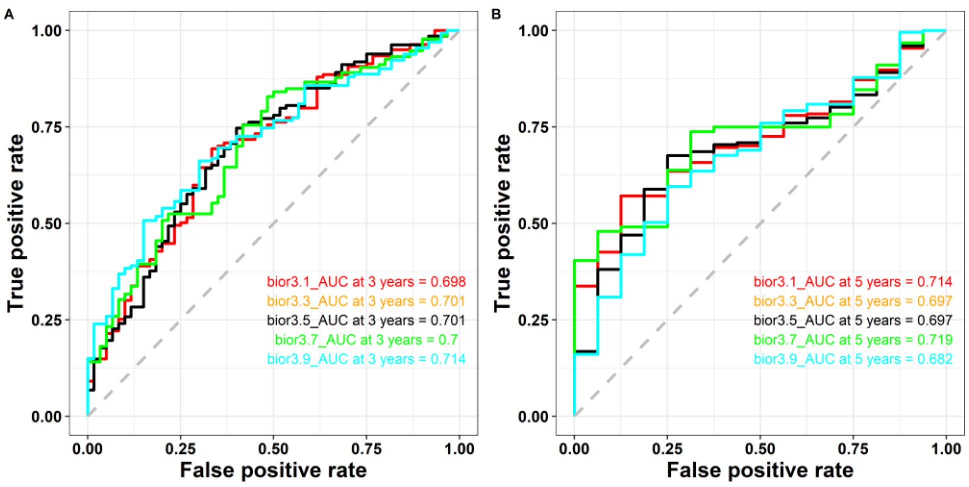

Figure 4. The performance of STAD dataset on bior basis function (bior3.1~bior3.9).

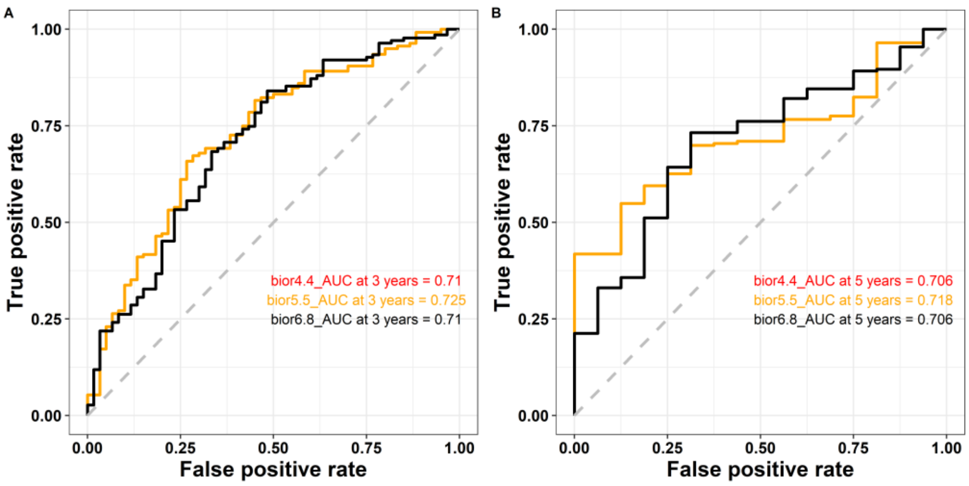

Figure 5. The performance of STAD dataset on bior basis function (bior4.4~bior6.8).

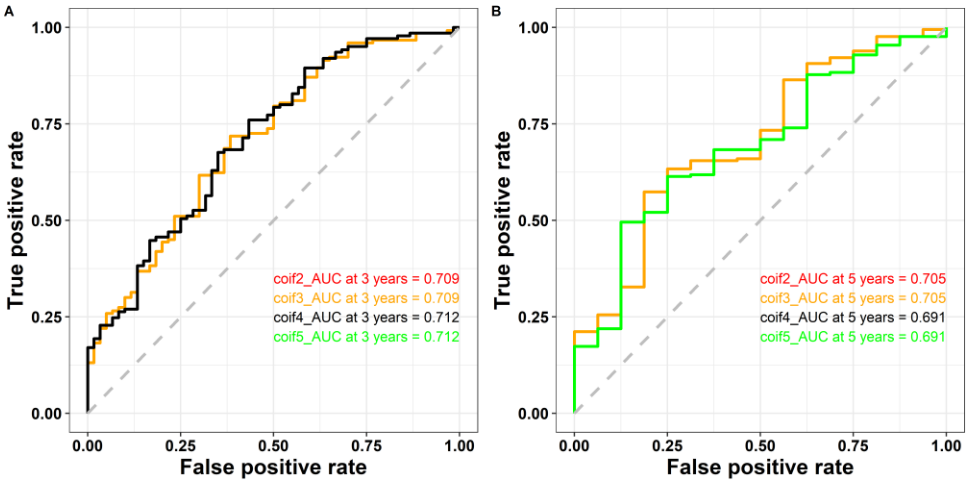

Figure 6. The performance of STAD dataset on coif basis function.

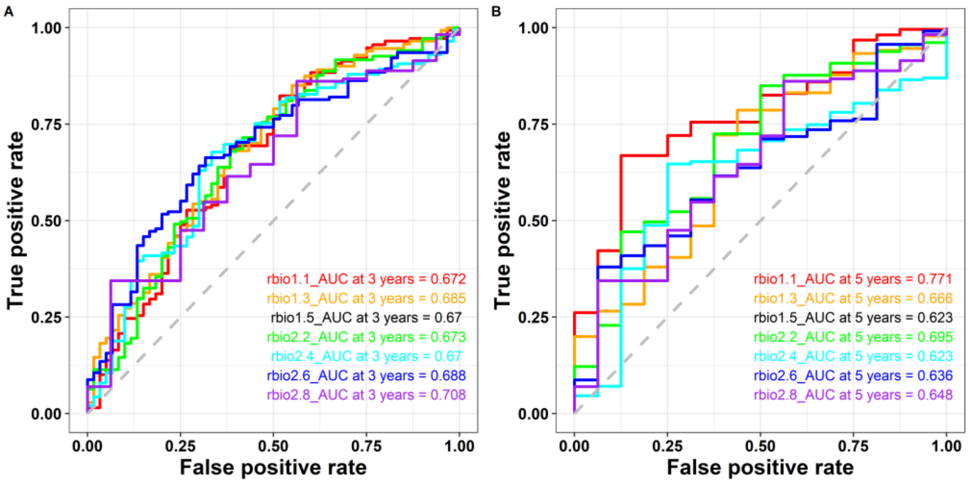

Figure 7. The performance of STAD dataset on rbio basis function (rbio1.1~rbio2.8).

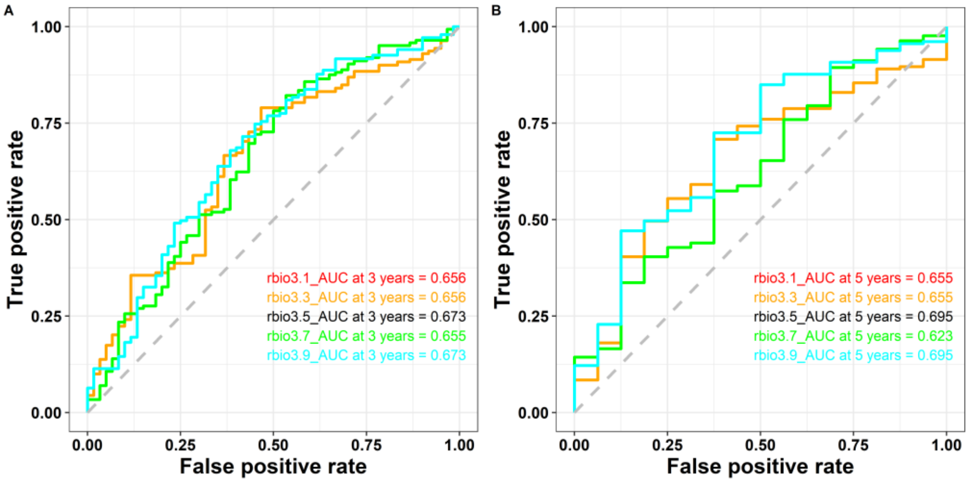

Figure 8. The performance of STAD dataset on rbio basis function (rbio3.1~rbio3.9).

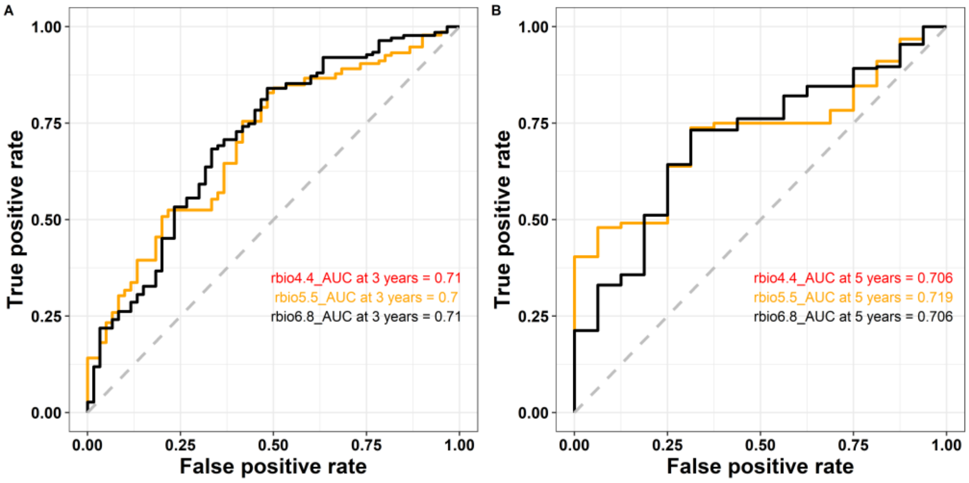

Figure 9. The performance of STAD dataset on rbio basis function (rbio4.4~rbio6.8).

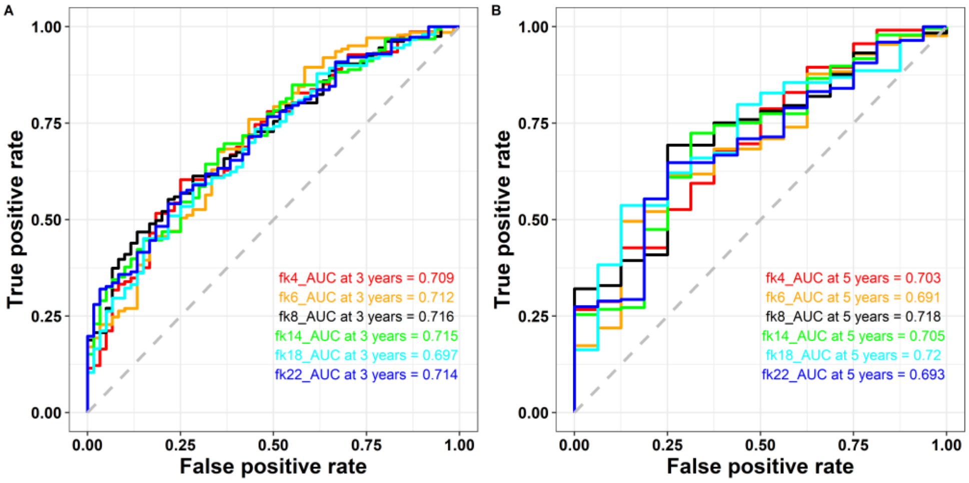

Figure 10. The performance of STAD dataset on fk basis function.

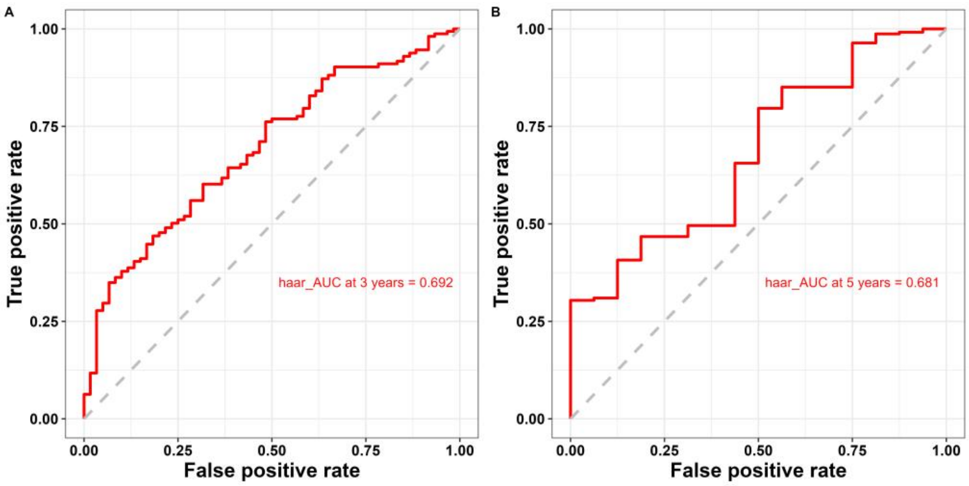

Figure 11. The performance of STAD dataset on haar basis function.

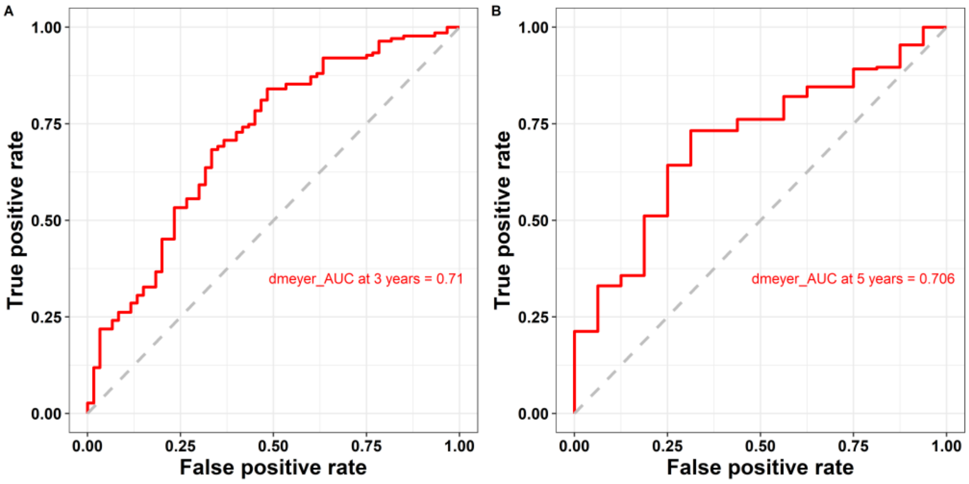

Figure 12. The performance of STAD dataset on dmeyer basis function.

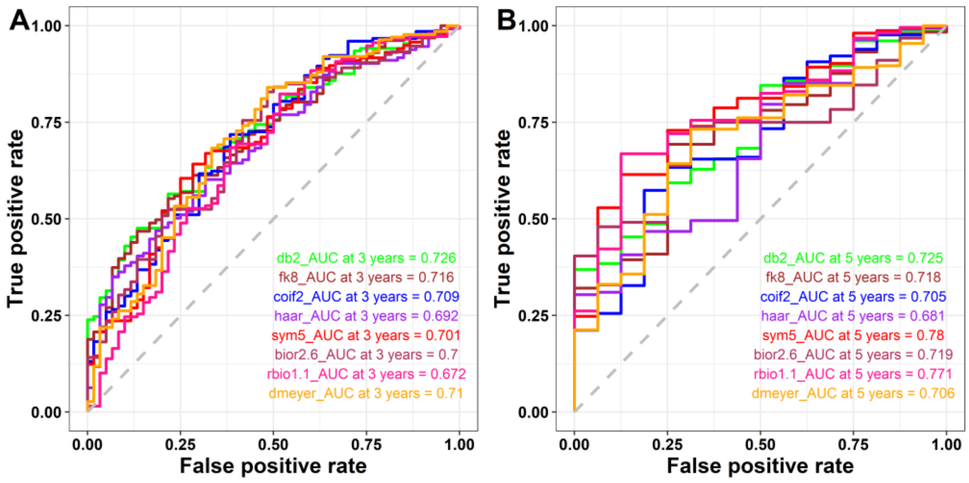

Figure 13. The performance of STAD dataset on all basis functions (Take the smoothness corresponding to the best result for each basis function).

3. Comparison of the three methods

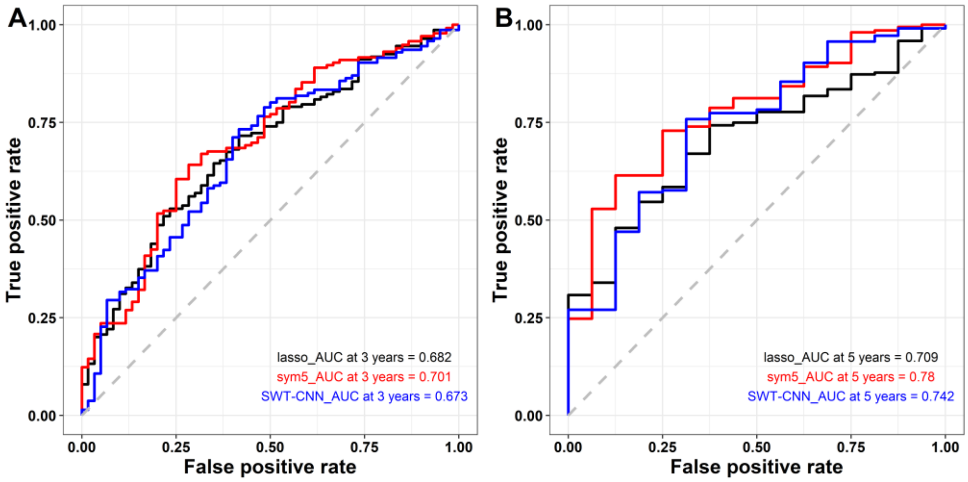

Figure 16. Comparison of the three methods on the STAD dataset. (A) AUC at 3 years: sym5_AUC at 3 years = 0.701, lasso_AUC at 3 years = 0.682, SWT-CNN at 3 years = 0.673; (B) AUC at 5 years: sym5_AUC at 5 years = 0.780, lasso_AUC at 5 years = 0.709, SWT-CNN at 5 years = 0.742.
